# Vehicle Trajectory Prediction Algorithm Based on Hybrid Prediction Model with Multiple Influencing Factors

**DOI:** 10.3390/s25041024

**Published:** 2025-02-09

**Authors:** Tao Wang, Yiming Fu, Xing Cheng, Lin Li, Zhenxue He, Yuchi Xiao

**Affiliations:** 1 School of Information and Communication Engineering, Beijing Information Science and Technology University, Beijing 100101, China; wt860122@buaa.edu.cn (T.W.); cheng@bistu.edu.cn (X.C.); 2023020575@bistu.edu.cn (Y.X.); 2 Key Laboratory of Photoelectric Testing Technology and Instruments, Beijing Information Science and Technology University, Beijing 100192, China; 3 National Computer Network Emergency Response Technical Team/Coordination Center of China, Beijing 100029, China; linl.ahch@buaa.edu.cn; 4 Hebei Provincial Key Laboratory of Agricultural Big Data, Hebei Agricultural University, Baoding 071001, China; hezhenxue@buaa.edu.cn

**Keywords:** autonomous driving, trajectory prediction, LSTM, multi-head attention

## Abstract

In the domain of autonomous driving systems, vehicle trajectory prediction represents a critical aspect, as it significantly contributes to the safe maneuvering of vehicles within intricate traffic environments. Nevertheless, a preponderance of extant research efforts have been chiefly centered on the spatio-temporal relationships intrinsic to the vehicle itself, thereby exhibiting deficiencies in the dynamic perception of and interaction capabilities with adjacent vehicles. In light of this limitation, we propose a vehicle trajectory prediction algorithm predicated on a hybrid prediction model. Initially, the algorithm extracts pertinent context information pertaining to the target vehicle and its neighboring vehicles through the application of a two-layer long short-term memory network. Subsequently, a fusion module is deployed to assimilate the characteristics of the temporal influence, spatial influence, and interactive influence of the surrounding vehicles, followed by the integration of these attributes. Ultimately, the prediction module is engaged to yield the predicted movement positions of the vehicles, expressed in coordinate form. The proposed algorithm was trained and validated using the publicly accessible datasets I-80 and US-101. The experimental results demonstrate that our proposed algorithm is capable of generating more precise prediction results.

## 1. Introduction

In recent years, with the advancement of sensor technology, the number and types of sensors equipped on intelligent vehicles have increased significantly, thereby greatly promoting the development of intelligent driving technology. In the traditional vehicle driving process, various sensor data could only play an auxiliary role, and drivers still needed to make corresponding judgments by combining visual information and the information fed back by sensors. To achieve the goal of intelligent driving, vehicles need to rely on sensor information to make independent decisions, such as by using cameras and LiDAR. Trajectory prediction serves as a critical connection within the realm of environmental information collection and decision making. The precision of the trajectory prediction directly influences the rationality of the subsequent decision making [1]. Currently, some enterprises have already provided relatively feasible solutions. For example, Baidu Apollo autonomous vehicles use the Inter-TNT model for prediction [2]. However, in complex scenarios with a large number of vehicles, the intentions of different vehicles may influence each other. How to predict vehicle trajectories with high efficiency and accuracy remains a well-recognized, difficult problem in this field [3].

In some relatively simple scenarios (for example, on a road without other vehicles or obstacles), the future trajectory of a vehicle is largely derived from its own historical trajectory. Since it is not interfered with by other factors, the behavior patterns it adopts are also easy to statistically analyze and summarize. In such scenarios, the time-based influence of the vehicle itself is the main factor to be considered in trajectory prediction. Therefore, even relatively simple prediction models can achieve good results, such as solutions based on classical physical models and machine learning solutions. Among them, most schemes based on classical physical models regard vehicle behaviors as pure physical motions. The physical models used include the uniform acceleration model [4], the model based on Kalman filtering [5], the model based on Monte Carlo [6], etc. However, due to the large number of interference factors affecting vehicle intentions in complex traffic environments, these classical physical models are only effective within a very short period of time (usually less than 1 s), which is obviously far from sufficient. With the continuous progress of theories of machine learning, schemes based on machine learning have gradually become mainstream. Compared with classical physical models, schemes based on machine learning can exhibit better prediction effects and have also enabled vehicle trajectory prediction capabilities to grow from short-term prediction to prediction in simple driving scenarios [7], for example, by using Support Vector Machines (SVMs) [8], Hidden Markov Models [9], etc. However, as driving scenarios become more complex, simple machine learning schemes can no longer meet the needs in complex traffic scenarios. Therefore, scholars in this field have shifted their attention to more advanced and complex machine learning models, such as deep learning.

When there are many vehicles around, the distance between vehicles also becomes an important factor in trajectory prediction, which is also an additional part considered by many of the current prediction models. For example, when the distance between vehicles is too close, drivers usually take actions such as decelerating or giving way. Essentially, trajectory prediction belongs to time series prediction. As one of the solutions capable of addressing long-term dependence issues, the Recurrent Neural Network (RNN) is one of the popular research directions in the field of trajectory prediction. In Reference [10], a hybrid trajectory prediction framework encoded by long short-term memory (LSTM) is proposed to evaluate the impact of a vehicle’s own behavior on surrounding vehicles. It introduces a reactive social convolution structure, which was designed to simulate the planned trajectory of the ego vehicle and the historical trajectories of surrounding vehicles. By doing so, it aims to mitigate the uncertainty of potential trajectories and effectively reduce the errors in trajectory prediction. Reference [11] puts forward a vehicle trajectory prediction model grounded in multivariate interaction modeling. By modeling the dynamic interactions among vehicles to obtain and fuse interaction information, while simultaneously using LSTM to construct a prediction module, the accuracy of the trajectory prediction was effectively improved. Reference [12] puts forward a novel lane-change decision model that caters to long-term prediction requirements. This model employs a long-term trajectory prediction model grounded in LSTM to predict the trajectories of surrounding vehicles. Additionally, the authors established a lane-change decision model based on fuzzy inference to infer the relationships between the target vehicle and other vehicles. In Reference [13], the authors started from improving the prediction accuracy and propose a distributed, decoupled long short-term memory (LSTM) self-trajectory prediction method. It uses a decoupling gate and a control gate to construct parallel LSTM cells and utilizes parallel cells to establish a distributed network architecture, substantially enhancing the accuracy of vehicle trajectory prediction.

Besides the RNN and its corresponding variants, the emergence of Transformer has also attracted researchers’ attention to the attention mechanism [14,15,16,17,18]. Consequently, certain researchers have endeavored to extend the application of this approach to the domain of trajectory prediction and have attained notable outcomes. Reference [19] puts forward a collaborative vehicle positioning and trajectory prediction framework grounded in belief propagation and the Transformer model. In this framework, a factor graph is meticulously constructed based on the sensor measurement values transmitted by vehicles. Subsequently, an enhanced belief propagation process is employed to approximate the posterior distribution of vehicles. Moreover, hidden features are extracted from historical position and vehicle motion data to model the long-term and short-term motion patterns of vehicles. Although researchers have made great efforts in vehicle trajectory prediction, the current achievements still have certain limitations. Most of the existing neural network-based methods start from the time-based influence of vehicles and the spatial distance influence between vehicles, such as the historical trajectory of the vehicle itself, the historical trajectories of surrounding vehicles, and the historical positions and spatial distances between vehicles. Although these factors are sufficient to show a good performance in ordinary traffic scenarios, they are still insufficient for complex traffic scenarios. To further improve the prediction accuracy in complex traffic scenarios, more influencing factors need to be taken into account.

Faced with diverse driving environments and complex and changeable collected information, some commonly used machine learning models may gradually fail to meet the requirements. Some researchers choose to construct suitable frameworks and models on their own according to specific application scenarios and application requirements. Reference [20] considers the perception failure situations that occur during the autonomous driving process and proposes a prediction-oriented perception enhancement framework to improve the performances of the existing interaction-based trajectory prediction models in the face of vehicle and sensor failures in the real world and could effectively reduce unreasonable trajectory inputs. Reference [21] shifts its focus to the problems commonly existing in prediction methods, such as deployment difficulties, insufficient computing resources, and error propagation, and specifically proposes a new multi-task parallel joint framework. Based on original LiDAR data, it conducts vehicle detection, state assessment, tracking, and trajectory prediction simultaneously. This approach effectively enhances the capabilities of the model. Reference [22] puts forward a greedy trajectory prediction method for autonomous vehicles. This method is grounded in the discrete-time-dimensional Gaussian process–information entropy probability framework, along with the hazard index map. Through this framework, it aims to estimate the intentions of surrounding vehicles and carry out trajectory prediction.

In recent years, the development of reinforcement learning techniques and generative models has also provided new ideas for vehicle trajectory prediction. Reference [23] elaborates on the basic principles of reinforcement learning and its applications in fields such as vehicle trajectory prediction and autonomous driving. It summarizes and deeply discusses reinforcement learning algorithms in recent years, such as imitation learning and deep Q-learning. Reference [24] combines the attention mechanism with inverse reinforcement learning. By using a social attention model based on the distance between vehicles to generate feature vectors and determine the weights of the attention mechanism, more surrounding vehicles can be taken into account. Reference [25] proposes a more generalized, secure, and robust hierarchical reinforcement learning framework. It adopts hierarchical double-deep Q-learning combined with LSTM to mitigate the impacts of noise and dynamic driving behaviors, thereby obtaining a more stable and versatile model.

In terms of generative models, Reference [26] presents a GAN model based on LSTM, where the GAN module can use a generative adversarial network to obtain the predicted trajectories of pedestrians. Although it is not specifically for vehicles, it can still provide new inspiration for vehicle trajectory prediction. Considering the problem of the insufficient extraction of vehicle hidden states by the social generative adversarial network, Reference [27] proposes an attention generative adversarial network. It uses the attention mechanism to focus on the weights of the influence of surrounding vehicles on the target vehicle and then conducts joint training with the generative adversarial network to generate predicted trajectories that meet the constraints. Reference [28] proposes a context-aware method, ContextVAE, for multi-modal vehicle trajectory prediction. Based on a time-varying auto-encoder, it uses a dual-attention mechanism to simultaneously focus on the environmental context and the states of dynamic agents, thereby providing accurate trajectory predictions. Although reinforcement learning and generative model techniques can produce more detailed and accurate results compared with other prediction methods, these techniques require a large amount of time and computational resources for training.

**Figure 1:**
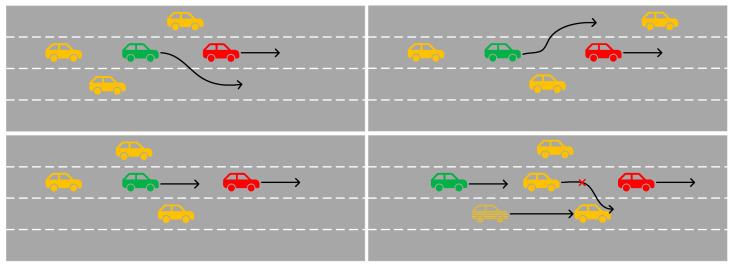
The trajectories of vehicles in different scenarios. The green ones represents the target vehicles, the yellow ones indicate those with the same speed as the main vehicle, and the red ones stand for those with a higher speed than the target vehicle. The black arrow represents the next driving route and direction of the vehicle.

In complex traffic scenarios, the intentions of drivers may also be affected by the behaviors of other vehicles. Figure 1 shows several possible lane situations. In the scenario in the lower-right corner, the vehicle in front of the target vehicle is overtaking, but the fast-approaching vehicle in the adjacent lane makes that vehicle give up the intention of overtaking and return to its original lane. During this process, the target vehicle may switch from a decelerating state (providing enough space for the vehicle in front to overtake) to an accelerating state (occupying the space left after the vehicle in front overtakes), and then quickly switch back to a decelerating state (providing enough space for the vehicle in front that gives up overtaking). As the traffic scenarios become more complex, there will be more and more factors affecting the vehicle trajectories. In order to effectively improve the accuracy of vehicle trajectory prediction and the utilization rate of sensor data, it is very necessary to consider more influencing factors.

By synthesizing the achievements of researchers in related fields, in this study, we finally selected three main influencing factors to form a set of multiple influencing factors, namely, the time-based influence, spatial influence, and interaction influence of surrounding vehicles. Through a comprehensive analysis of these factors, the model can better understand vehicle behaviors. Compared with the existing neural network-based methods, this paper additionally adds the factor of the interaction influence of surrounding vehicles, which helps the model to more accurately predict the future trajectory of the target vehicle. In order to fully calculate the influence of time, space, and the motion state on vehicle trajectories, this paper proposes a vehicle trajectory prediction algorithm based on a hybrid prediction model on the basis of these multiple influencing factors. Firstly, the algorithm utilizes the feature extraction module based on the two-layer long short-term memory (LSTM) network to obtain the corresponding context information. Subsequently, the fusion module is employed to conduct an in-depth exploration of the features and fuse the obtained features. Finally, the fused features are output to the prediction module based on LSTM to directly output multi-step predictions in the future, that is, the predicted trajectories of the target vehicle. The main contributions of this paper are as follows:

1. A hierarchical feature extraction module is proposed to learn the influencing factors that may change the future trajectories of vehicles in complex driving scenarios, including the impact of the time, spatial distance, and motion patterns. Through comparative analysis, it can be seen that this method can effectively improve the accuracy of trajectory prediction;

2. A fusion module for mining and integrating features is proposed. Combined with social tensors, it extracts the hidden deep features in common spatial distances and motion patterns and fuses these features into mixed impact features so that the fusion module can capture multiple influencing factors;

3. A prediction module is proposed. It only adopts a one-step forward process to directly output multi-step predictions in the future. While ensuring the prediction effect, it speeds up the inference speed, enabling it to meet the requirements of real-time prediction.

The structure of the rest of this paper is as follows: Section 2 describes in detail the hybrid prediction model proposed in this paper. Section 3 presents the experimental results and discusses the performance differences among the models. Section 4 is the conclusion.

## 2. Materials and Methods

Before introducing the hybrid prediction model, it is necessary to analyze the trajectory prediction scenarios first. We regard the entire lane as a plane and establish a coordinate system, and the predicted coordinates of the target vehicle follow a bivariate Gaussian distribution. Then, the coordinates of all vehicles within a certain time period (t) can be expressed as follows:

(1) $$P=\{L_{1},L_{2},\cdots ,L_{t}\}$$

(2) $$L_{t}=[{\left(x_{t}^{1},y_{t}^{1},vx_{t}^{1},vy_{t}^{1},ax_{t}^{1},ay_{t}^{1}\right)}^{T},\cdots ,{\left(x_{t},y_{t},vx_{t},vy_{t},ax_{t},ay_{t}\right)}^{T},\cdots ,{\left(x_{t}^{N},y_{t}^{N},vx_{t}^{N},vy_{t}^{N},ax_{t}^{N},ay_{t}^{N}\right)}^{T}]$$

**Figure 2:**
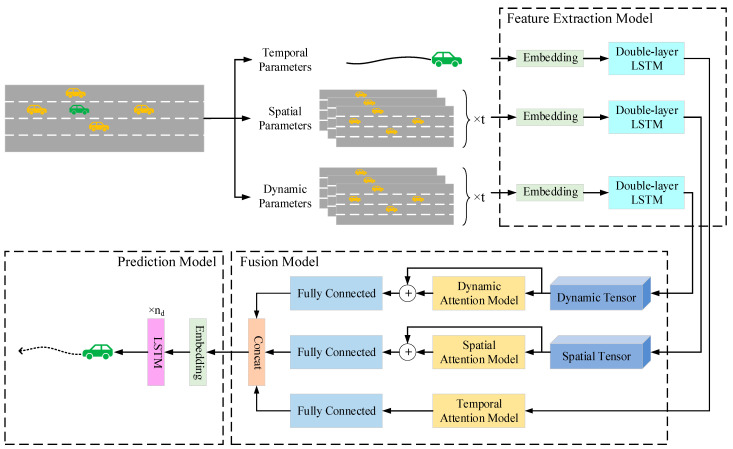
The structure diagram of the hybrid prediction model. The green ones represents the target vehicles, the yellow ones indicate those with the same speed as the main vehicle.

Then, the trajectory that needs to be predicted in the future can be expressed as follows:

(3) $$Y=[{\left({\hat{x}}_{t+1},{\hat{y}}_{t+1},\hat{v}x_{t+1},\hat{v}y_{t+1},\hat{a}x_{t+1},\hat{a}y_{t+1}\right)}^{T},\cdots ,{\left({\hat{x}}_{t+T},{\hat{y}}_{t+T},\hat{v}x_{t+T},\hat{v}y_{t+T},\hat{a}x_{t+T},\hat{a}y_{t+T}\right)}^{T}]$$

where P represents the parameter set of all vehicles in the past t time, $L_{t}$ represents the parameter set of all vehicles at time t, ${\left(x_{t},y_{t},vx_{t},vy_{t},ax_{t},ay_{t}\right)}^{T}$ represents the parameter set of the target vehicle. $x_{t}$ is the abscissa, $y_{t}$ is the ordinate, $vx_{t}$ represents the lateral velocity, $vy_{t}$ represents the longitudinal velocity, $ax_{t}$ represents the lateral acceleration, and $ay_{t}$ represents the longitudinal acceleration. N is the total number of other vehicles. Since we only focus on the future trajectory of the target vehicle, the output (Y) we want to obtain is all the coordinate information of the target vehicle within the future T time. Therefore, the vehicle trajectory prediction problem can be simplified to inputting the historical trajectories of the target vehicle and its surrounding vehicles and outputting the predicted trajectories in the future. Based on this, this paper proposes a hybrid prediction model, and the framework of this model is shown in Figure 2. It mainly consists of three parts: a feature extraction module, which is used to extract the relevant features of the target vehicle and its surrounding vehicles; a fusion module, which is used to extract the hidden time impact features, spatial distance impact features, and motion pattern impact features and fuse them; a prediction module, which is used to output the predicted trajectory of the target vehicle.

### 2.1. Feature Extraction Module

**Figure 3:**
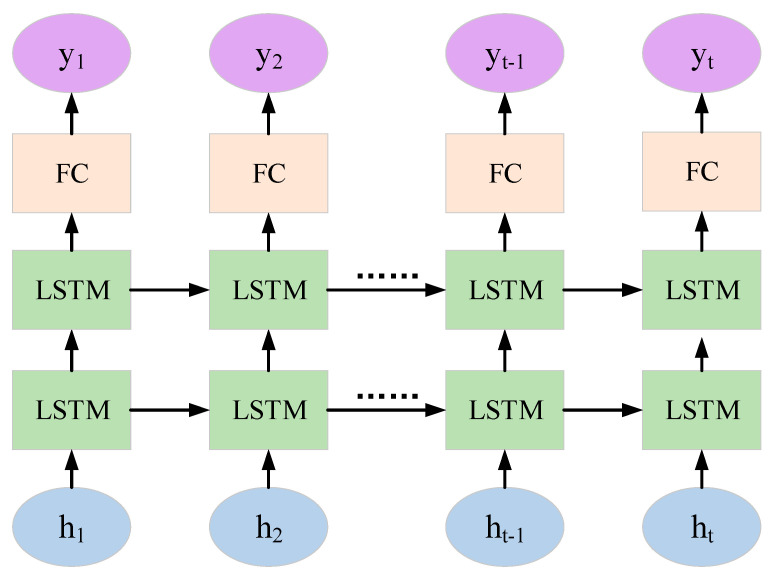
The structure diagram of the double-layer long short-term memory network.

This module consists of three embedding layers and multiple two-layer long short-term memory (LSTM) networks. Each embedding layer is composed of three parallel, fully connected layers, which are respectively responsible for processing the time-related parameters of the target vehicle (such as the coordinates, velocity, and acceleration), the spatial distance parameters of the surrounding vehicles, and the motion state parameters of the surrounding vehicles. The activation function chosen is LeakyReLU. The calculation methods of velocity and acceleration are as follows:

(4) $$v_{x_{t}}=\frac{x_{t+1}-x_{t}}{1/f},v_{y_{t}}=\frac{y_{t+1}-y_{t}}{1/f}$$

(5) $$a_{x_{t}}=\frac{v_{x_{t+1}}-v_{x_{t}}}{1/f},a_{y_{t}}=\frac{v_{y_{t+1}}-v_{y_{t}}}{1/f}$$

where $v_{x_{t}}$ and $v_{y_{t}}$ are the lateral and longitudinal velocities of the vehicle at time (t), $a_{x_{t}}$ and $a_{y_{t}}$ are the lateral and longitudinal accelerations of the vehicle at time (t), and f is the sampling frequency. Since vehicle trajectory prediction belongs to long-distance dependence, traditional recurrent neural networks have difficulty dealing with it. Therefore, we adopt an improved recurrent neural network, namely, the long short-term memory (LSTM) network. It introduces a unique gate control mechanism, enabling it to retain information from a long time ago while also preventing insignificant content from entering the memory. Moreover, the multi-dimensional hidden outputs of the multiple time steps in the first layer of the two-layer LSTM are used as the time step inputs for the second layer, thereby helping the model capture more complex sequence features and enhancing the depth and learning ability of the model. Consequently, this paper selects the two-layer LSTM as the backbone of the feature extraction module and inputs the feature information processed by the embedding layer to extract hidden features. Its specific structure is shown in Figure 3.

### 2.2. Fusion Module

**Figure 4:**
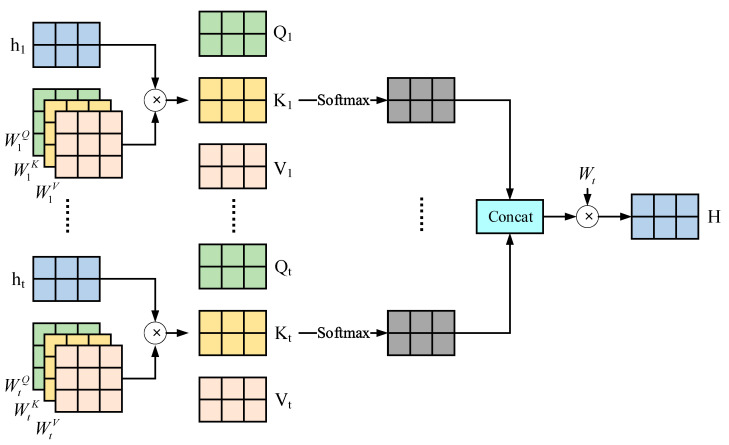
The structure diagram of the multi-head attention.

Since we need to take more influencing factors into account in complex scenarios, we set up a fusion part to fuse multiple different features. The specific structure is shown in Figure 4. Firstly, the states of the target vehicle at different moments will have a certain impact on the trajectory [29]. It is crucial to effectively capture and analyze this temporal impact. Therefore, we choose the multi-head attention mechanism to construct a time attention module so as to learn the impact of time on vehicles. The attention mechanism captures and learns the weights of vehicles at different moments, and the multi-head attention mechanism enables the model to pay attention to information at different positions simultaneously and improves the stability of the model.

We input the $H_{t}$ extracted from the target vehicle parameters in the previous part into the time attention module and then obtain the time impact feature ($H_{T}$). The specific calculation formula is as follows:

(6) $$\left\{\begin{array}{l} Q_{Ti}=\phi (H_{ti},W_{i}^{Q})\\ K_{Ti}=\phi (H_{ti},W_{i}^{Q})\\ V_{Ti}=\phi (H_{ti},W_{i}^{Q}) \end{array}\right.$$

(7) $$head_{i}=\mathrm{softmax}(\frac{Q_{Ti}K_{Ti}^{T}}{\sqrt{d_{K}}})V_{Ti}$$

(8) $$H_{T}=f(\phi (\mathrm{Concat}(head_{1},head_{2},\cdots ,head_{t})W^{H},W_{T}))$$

where $Q_{Ti}$, $K_{Ti}$, and $V_{Ti}$ are the query matrix, key matrix, and value matrix of the scaled dot-product attention corresponding to the *i*-th attention head in the time attention module, respectively. $d_{K}$ is the scaling factor, $H_{T}$ is the finally obtained time impact feature, and the parameters starting with W are all trainable parameters. Apart from time, the spatial distances between vehicles and the motion patterns of surrounding vehicles are also important factors influencing the trajectories. In the traditional driving process, these two parameters are also the main bases for drivers to make decisions. For this reason, in this part, we add social tensors on the basis of the multi-head attention mechanism to extract deeper features. The social tensor was originally proposed as S-LSTM, aiming to analyze the intentions and interactions among pedestrians, and it was adjusted and applied to the vehicle field in CS-LSTM [30]. Specifically, we construct the tensor ($H_{nl\_tensor}$) that describes the spatial distance and the tensor ($H_{nd\_tensor}$) that describes the vehicle motion state, respectively, according to the $H_{nl}$ and $H_{nd}$ obtained in the feature extraction module. The specific formulas are shown as follows:

(9) $$H_{nl\_tensor}=\sum_{i\in N}I(x_{i},x)H_{nl},H_{nd\_tensor}=\sum_{i\in N}I(x_{i},x)H_{nd}$$

where $X_{i}$ and X are the coordinate matrices of the other vehicle (i) and the target vehicle, respectively, I() is the indicator function, and N is the total number of other vehicles. After that, the obtained tensors are respectively passed into the spatial distance attention module and the motion pattern attention module, which are similar to the time attention module, so as to extract the hidden features $H_{nl}^{\prime }$ and $H_{nd}^{\prime }$ in the tensors. Considering that this part needs to extract deeper features in order to alleviate the possible vanishing gradient problem, we also add residual connections. Eventually, we can obtain the spatial distance impact feature and the motion pattern impact feature:

(10) $$\left\{\begin{array}{l} H_{NL}=f(\phi (H_{nl}^{\prime },W_{NL}))+H_{nl\text{\_}tensor}\\ H_{ND}=f(\phi (H_{nd}^{\prime },W_{ND}))+H_{nd\text{\_}tensor} \end{array}\right.$$

By fusing the three types of features obtained from the above processes, the mixed impact feature can be obtained:

(11) $$H_{mix}=\mathrm{Concat}(H_{T},H_{nl},H_{nd})$$

### 2.3. Prediction Module

**Figure 5:**
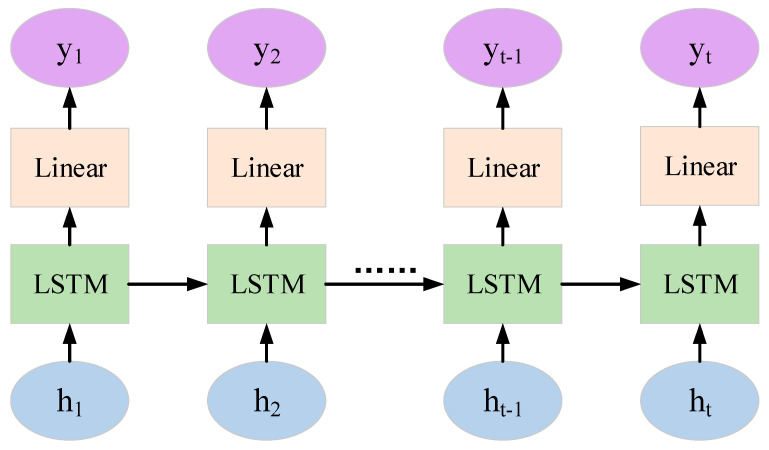
The structure diagram of the direct multi-step prediction model.

Currently, most long time series predictions based on LSTM adopt the Seq2Seq method. This method uses an encoder–decoder architecture to process time series data. The encoder encodes the input sequence into a fixed-length vector, and then the decoder uses this vector to generate the output sequence. This method can capture the long-term dependence relationships in the sequence, but it is rather difficult to train and slows down the inference speed of the model. Moreover, since this method outputs the previous prediction time step together with the hidden state to the current prediction time step, it will also lead to an increase in the network error. Therefore, we adopt the direct multi-output method, as shown in Figure 5. This method directly outputs multi-step predictions in the future with only one forward step, saving the time for step-by-step iterative inference. The specific formula is shown as follows:

(12) $$Output=\phi (LSTM(f(\phi (H_{mix},W_{1})),W_{2}),W_{3})$$

where LSTM() represents all the operations included in the LSTM network, and the parameters starting with W are all trainable parameters. Specifically, this module first processes the time dimension of the obtained mixed impact features, converts it to the target prediction time length (T), and then inputs it into the LSTM network and outputs the corresponding prediction results. Finally, the entire hybrid prediction model is trained by minimizing the negative log-likelihood loss while updating the parameters of the model:

(13) $$Loss=-\sum_{i=t+1}^{t+T}\log(P(x_{i},y_{i}|{\hat{\mu }}_{i},{\hat{\sigma }}_{i},{\hat{\rho }}_{i}))$$

where ${\hat{\mu }}_{i}$, ${\hat{\sigma }}_{i}$, and ${\hat{\rho }}_{i}$ are the mean, standard deviation, and correlation coefficient, respectively.

## 3. Experiment Results and Discussion

### 3.1. Datasets

To evaluate the hybrid prediction model proposed in this paper, we used two parts, namely, I-80 and US-101, from the Next Generation Simulation (NGSIM) dataset of highway driving in the United States collected by the Federal Highway Administration (FHWA) [31]. This dataset records the driving conditions of all vehicles on specified roads within a certain period of time. The specific time periods are from 4:00 pm to 4:15 pm, from 5:00 pm to 5:15 pm, and from 5:15 pm to 5:30 pm, totaling 45 min. The sampling frequency of the data is 10 Hz, and the data include information such as vehicle coordinates and speeds. It can effectively restore traffic scene information under different levels of complexity and simultaneously verify the robustness of the algorithm proposed in this paper.

Compared with the commonly used KITTI dataset and NuScenes dataset [32], the most prominent feature of the NGSIM dataset is that its data are collected by high-precision sensors installed at road scenes, rather than from the perspective of the vehicle itself. Therefore, for complex traffic scenarios, the NGSIM dataset based on the road segment perspective can provide a more objective and accurate data source for vehicle trajectory prediction research.

In the selected parts of I-80 and US-101, 70% of the content was used as the training set, and 30% was used as the test set. Regarding the pre-processing scheme, since we needed to compare it with other neural network schemes, to avoid errors in the final experimental results caused by inconsistent pre-processing steps, we followed the processing scheme proposed by Nachiket Deo et al. in Reference [30]. In this scheme, the original data are downsampled to 5 Hz, and the duration of each trajectory is cropped to 8 s. Among these data, the first 3 s are used as the historical trajectory to extract relevant information, and the subsequent 5 s are used for comparison with the prediction results, so as to evaluate the error between the predicted results and the actual results.

### 3.2. Evaluation Metrics

In the evaluation stage, we selected three commonly used evaluation metrics in the field of vehicle trajectory prediction, namely, the average displacement error (*ADE*) [33], final displacement error (*FDE*) [33], and root mean square error (*RMSE*) [34]. The calculation methods are shown as follows:

(14) $$ADE=\frac{1}{N}\sum_{i=1}^{N}\sqrt{{\left({\hat{x}}_{i}-x_{i}\right)}^{2}+{\left({\hat{y}}_{i}-y_{i}\right)}^{2}}$$

(15) $$FDE=\sqrt{{\left({\hat{x}}_{t+N}-x_{t+N}\right)}^{2}+{\left({\hat{y}}_{t+N}-y_{t+N}\right)}^{2}}$$

(16) $$RMSE=\sqrt{\frac{1}{N}\sum_{i=1}^{N}\left[{\left({\hat{x}}_{i}-x_{i}\right)}^{2}+{\left({\hat{y}}_{i}-y_{i}\right)}^{2}\right]}$$

**Figure 6:**
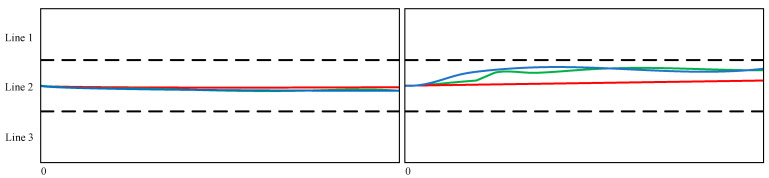
Visualization of prediction result display. For the convenience of viewing, we connected the predicted points, drew the corresponding prediction curves, and cropped the starting point of the target vehicle to the leftmost side. Among them, the black dotted line represents the lane line, the blue one is the real trajectory, the green one is the prediction trajectory given by our model, and the red one is the prediction trajectory given by the CS-LSTM model.

In terms of the performance comparison, we chose three models, namely, V-LSTM [30], S-LSTM [35], and CS-LSTM [30]. Among them, V-LSTM only conducts trajectory prediction based on the time impact of the target vehicle itself and uses LSTM for trajectory prediction, yet it lacks the analysis of the influence of surrounding vehicles. S-LSTM transfers the pedestrian trajectory prediction method to the vehicle trajectory prediction field and also has the problem of insufficient analysis of the time impact on the target vehicle itself to some extent. CS-LSTM is an improvement based on S-LSTM, replacing the fully connected layer with a convolutional pooling network. The performances of the different models are shown in Table 1, Table 2 and Table 3. The visualization results are shown in Figure 6.

### 3.3. Analysis

The hybrid prediction model proposed in this paper is implemented within the Python programming language and the PyTorch deep learning platform framework. To simplify the analysis, the research scope of this paper was set to the two lanes adjacent to the target vehicle, and the lengths of both the front and rear lanes were set to 90 feet. During the training process, we used the Adam optimizer with a learning rate of 0.001, the activation function was Leaky ReLU, and the batch size was 128. Regarding the specific parameters of the model, in the feature extraction module, the fully connected layer expands the dimension of the original features to 32, and the number of layers of the double-layer LSTM is set to 2. In the fusion module, the number of heads of the three attention modules is set to 3. In the prediction module, the fully connected layer expands the time dimension to 25, the number of layers of the LSTM is set to 1, and the number of hidden units is 128.

From the perspective of the root mean square error (RMSE), as the simplest model, the RMSE of the V-LSTM is much larger than those of the other three methods. Even compared with the S-LSTM, which has the second-worst performance, the gap reaches 34%. This indicates that only considering the time impact of the vehicle itself is very inaccurate and also shows that surrounding vehicles have a relatively obvious impact on the future trajectory of the target vehicle. S-LSTM and CS-LSTM, which take into account both the spatial interaction and time effect simultaneously, exhibit relatively higher accuracies. However, they still fall short compared to the model proposed in this paper. Compared with S-LSTM and CS-LSTM, the average RMSE values of our model are reduced by 9.7% and 6.1%, respectively.

Regarding the aspects of the average displacement error (ADE) and final displacement error (FDE), the accuracy of the trajectory prediction by S-LSTM and CS-LSTM has been significantly improved. Our model further considers the impact of the motion patterns among vehicles on this basis, so the accuracy has been improved to some extent. The indicators within all time ranges are the best, which also proves that the three factors influencing vehicle trajectory prediction proposed by us are reasonable and necessary, namely, the vehicle states at different moments, the spatial distances between vehicles, and the motion patterns of surrounding vehicles. Compared with the relatively better-performing CS-LSTM, the average displacement error of our model is reduced by 9.4%, and the average final displacement error is reduced by 7.3%.

### 3.4. Ablation Experiment

The model in this paper takes into account three different influencing factors, namely, the temporal influence, spatial influence, and surrounding vehicle interaction influence. To demonstrate the effectiveness of the newly added influencing factors, we additionally constructed two different variants of the model based on the same dataset. One is the model lacking the features of the surrounding vehicle interaction influence (abbreviated as Lack-SVI), and the other is the model directly using the Seq2Seq multi-step prediction module in the prediction stage (abbreviated as Seq2Seq). The comparison of the prediction results between these two variant models and those of our model is shown in Table 4. For ease of representation, in each table, the first number is the average displacement error (ADE), the second number is the final displacement error (FDE), and the third number is the root mean square error (RMSE).

It can be observed that in the initial period, the prediction performances of the three models were not significantly different. However, as the prediction time increased, the variant models gradually failed to fully capture the key information around the target vehicle, resulting in higher prediction errors. As the prediction time continued to increase, the prediction errors became larger and larger. This fully demonstrates the effectiveness of our adoption of three different influencing factors. They can accurately and comprehensively capture the surrounding feature information, thereby effectively reducing prediction errors and improving the prediction performance of the model.

In addition, to verify the rationality of the number of LSTM layers in the model, we modified the number of layers (n_1_) of the double-layer LSTM in the feature extraction module and the number of layers (n_2_) of the LSTM in the prediction module of the model. Then, we conducted corresponding tests in the same way as the ablation experiment of the variant models. The test results are shown in Table 5. Finally, the network configuration of the model was determined, that is, n_1_ = 2 and n_2_ = 1.

## 4. Conclusions

In the domain of intelligent driving, vehicle trajectory prediction has emerged as a prominent and highly challenging research area. Nevertheless, the current state of vehicle trajectory prediction research is marred by the issue of the inadequate consideration of influencing factors. The majority of the existing models predominantly base their predictions either on the state alterations of the vehicle per se or on the spatial elements in its vicinity, which may suffice for relatively uncomplicated driving scenarios. However, in the context of complex driving situations, the intentions of surrounding vehicles assume a crucial significance.

In light of this circumstance, this paper puts forward a vehicle trajectory prediction algorithm predicated on a hybrid prediction model. This algorithm comprehensively incorporates three influential factors: the vehicle states at diverse time instants, the spatial distances between vehicles, and the motion patterns of the surrounding vehicles. The objective is to address the problem of the suboptimal prediction accuracy prevalent in the field of vehicle trajectory prediction. We utilized publicly accessible datasets, namely, US-101 and I-80, for the training and subsequent analysis. Based on the obtained analysis results, it can be inferred that the model proposed herein demonstrates superiority over other comparative models in terms of three evaluation metrics, namely, the average displacement error, final displacement error, and root mean square error. This implies that the vehicle trajectory prediction algorithm proposed in this paper is capable of effectively augmenting the accuracy of trajectory prediction.

Nonetheless, the algorithm proposed in this paper is not without certain limitations. For instance, the model confines its consideration to vehicles as the principal entities, rendering it rather arduous for the management of various irregularly shaped obstacles (such as motorcycles and bicycles). Recently, graph neural networks have piqued our interest. They possess advantages such as taking neighbor information into account, weight sharing, and strong generalization capabilities, and are also applicable to vehicle trajectory prediction. Our next plan is to integrate graph neural networks with LSTM to conduct more in-depth research on the recognition of the vehicle’s surrounding environment and vehicle trajectory prediction [36].

## Figures and Tables

**Table 1:** Comparison of RMSEs among different models.

Metric (m)	Prediction Time (s)	V-LSTM	S-LSTM	CS-LSTM	Ours
RMSE	1	0.69	0.66	0.61	0.56
	2	1.66	1.30	1.25	1.19
	3	2.90	2.15	2.08	1.95
	4	4.48	3.27	3.12	2.90
	5	6.24	4.54	4.38	4.14
	Average	3.19	2.38	2.29	2.15

**Table 2:** Comparison of ADEs among different models.

Metric (m)	Prediction Time (s)	V-LSTM	S-LSTM	CS-LSTM	Ours
ADE	1	0.21	0.22	0.21	0.19
	2	0.53	0.45	0.43	0.41
	3	0.95	0.72	0.71	0.63
	4	1.39	1.03	1.01	0.91
	5	1.94	1.37	1.36	1.22
	Average	1.00	0.76	0.74	0.67

**Table 3:** Comparison of FDEs among different models.

Metric (m)	Prediction Time (s)	V-LSTM	S-LSTM	CS-LSTM	Ours
FDE	1	0.43	0.38	0.38	0.34
	2	1.19	0.91	0.89	0.83
	3	2.14	1.51	1.50	1.36
	4	3.33	2.29	2.25	2.09
	5	4.75	3.27	3.21	2.97
	Average	2.37	1.67	1.64	1.52

**Table 4:** The comparison results of the ablation experiment.

Prediction Time (s)	Lack-SVI	Seq2Seq	Ours
1	0.19/0.37/0.56	0.30/0.43/0.58	0.19/0.34/0.56
2	0.42/0.86/1.21	0.49/0.89/1.21	0.41/0.83/1.19
3	0.68/1.43/1.95	0.75/1.46/2.01	0.63/1.36/1.95
4	0.99/2.17/2.98	1.03/2.18/2.98	0.91/2.09/2.90
5	1.30/3.11/4.25	1.33/3.08/4.26	1.22/2.97/4.14

**Table 5:** Error comparison of different numbers of LSTM layers.

n_1_		1	2	3
	n_2_			
1		0.68/1.52/2.16	0.69/1.54/2.18	0.68/1.55/2.23
2		0.67/1.52/2.15	0.70/1.55/2.17	0.70/1.54/2.22
3		0.69/1.53/2.18	0.72/1.53/2.20	0.70/1.56/2.24

## Data Availability

Restrictions apply to the availability of these data. Data were obtained from the Federal Highway Administration and are available at https://data.transportation.gov/Automobiles/Next-Generation-Simulation-NGSIM-Vehicle-Trajector/8ect-6jqj (accessed on 24 November 2020) with the permission of the Federal Highway Administration.
